# Activation of GPR30 with G1 inhibits oscillatory shear stress-induced adhesion of THP-1 monocytes to HAECs by increasing KLF2

**DOI:** 10.18632/aging.202897

**Published:** 2021-04-19

**Authors:** Chi Chen, Jingyan Chen, Xuefei Tao, Minghuan Fu, Biao Cheng, Xiaohan Chen

**Affiliations:** 1 Department of Geriatrics, Sichuan Provincial People's Hospital, Affiliated Hospital of University of Electronic Science and Technology of China, Chengdu 610072, Sichuang, China

**Keywords:** oscillatory shear stress (OSS), atherosclerosis, endothelial cells, endothelial dysfunction, GPR30

## Abstract

Atherosclerosis is a chronic inflammatory disease known to be mediated by numerous factors, among which endothelial dysfunction plays a critical role. Oscillatory shear stress induces endothelial cells to lose their anti-atherosclerotic properties and downregulates the expression of the innate protective transcription factor, Krüppel-like factor 2 (KLF2), which is typically upregulated in vascular endothelial cells in response to harmful stimuli. Oxidative stress and inflammation impair endothelial function and damage their survival. Oscillatory shear stress also promotes generation of reactive oxygen species and production of pro-inflammatory cytokines such as tumor necrosis factor-α (TNF-α) and interleukin-6 (IL-6), thereby further promoting endothelial dysfunction and formation of atherosclerotic plaque. A major event in the development of atherosclerotic plaque is rolling and adhesion of monocytes to endothelial cells, which is mediated by adhesion molecules including vascular cellular adhesion molecule 1 and endothelial-selectin. Expression of these molecules is also upregulated by oscillatory shear stress. Estrogen has long been recognized as a protective agent against atherosclerosis, but the mechanisms through which estrogen receptors prevent atherogenesis remain unclear. In the present study, we investigated the role of the G-coupled protein estrogen receptor (GPR30) in oscillatory shear stress- induced endothelial dysfunction. We show that agonism of GPR30 by its specific agonist G1 prevented oscillatory shear stress -induced oxidative stress markers and production of inflammatory cytokines and adhesion molecules. As a result, GPR30 activation suppresses monocytes adhesion to endothelial cells. Furthermore, we demonstrate that GPR30 prevents oscillatory shear stress- induced downregulation of KLF2 via ERK5 pathway. These findings suggest that endothelial GPR30 is potential target to suppress oscillatory shear stress mediated atherogenesis.

## INTRODUCTION

Oscillatory shear stress (OSS) is recognized as a key contributor to the development of atherosclerosis as vascular endothelium plays a critical role on hemodynamics. OSS contributes to the initiation of an inflammatory signaling cascade, change of endothelial cell phenotype, and attachment of monocytes to endothelial cells triggered by the release of adhesion molecules [1–4]. Oxidative stress occurs when the amount of reactive oxygen species (ROS) exceed the regulatory capacity of the innate antioxidant system and triggers activation of macrophages and endothelial cells to produce proinflammatory cytokines, chemokines, and adhesion molecules. In vascular tissues, accumulation of ROS promotes immune cell infiltration and adhesion, platelet activation, and induce oxidation of lipids, proteins, and nucleic acid, thereby greatly contributing to atherogenesis [5]. Thus, remedy of oxidative stress is viewed as a potential treatment target for atherosclerosis [6]. As atherosclerosis is considered a chronic inflammatory disease, inhibiting the expression of proinflammatory cytokines such as interleukin-6 (IL-6) and IL-1β is another potential treatment strategy [7, 8]. Increased plasma levels of IL-6 are considered as an independent risk factor for atherosclerosis [9]. Monocyte chemoattractant protein 1 (MCP-1) is an important chemokine that drives recruitment of monocytes to the intima [10]. Recruited monocytes then roll along and adhere to the endothelial cells that comprise the intima, thereby generating atherosclerotic plaques. This process is enhanced by endothelial cell dysfunction and inflammation and is primarily governed by expression of cellular adhesion molecules, such as vascular cellular adhesion molecule 1 (VCAM-1) and endothelial-selectin (E-selectin) [11, 12]. In normal condition, expression of the transcriptional factor Krüppel-like factor 2 (KLF2) is increased in response to circulatory flow and induces a signal cascade involving diverse anti-inflammation, anti-thrombosis, and anti-oxidative stress mediators, thereby serving a protective role against atherogenesis [2]. However, KLF2 expression has been shown to be downregulated by disturbed shear stress, such as OSS [13]. Thus, restoring expression of KLF2 may serve as a potential treatment target against the atherogenic effects of OSS.

In recent years, the members of the G-coupled protein receptors (GCPR) family have received increasing attention for their roles in modulating diverse biological functions including inflammatory response and have been implicated in atherogenesis [14, 15]. The GCPR family is the largest membrane protein family in humans and modulation of GCPRs is considered a promising treatment target [16]. G-coupled protein receptor 30 (GPR30), also known as G-coupled protein estrogen receptor (GPER), is a Gs-coupled heptahelical transmembrane receptor that binds specifically with estrogens, both natural and analogues thereof, to activate adenylyl cyclase and transactivation of epidermal growth factor receptor (EGFR) [17]. Agonism of GPR30 has been shown to exert estrogen-dependent regulatory effects on vasomotor tone and protective effects against myocardial ischemia/reperfusion injury [18]. Since estrogen is a ligand for GPR30 but also ERα and ERβ, the highly specific GPR30 agonist (1-[4-(6-bromobenzo[1,3]dioxol-5yl)-3a,4,5,9b-tetrahydro-3H-cyclopenta [c]quinolin-8-yl]-ethanone) (G1) was developed in 2009 to differentiate GPR30-specific actions from those mediated by other ERs or GCPRs [19]. In the present study, we explored the effects of GPR30 agonism using G1 on markers of atherosclerosis by exposing human aortic endothelial cells (HAECs) to OSS (5 dyn/cm^2^) for various periods of time. Our findings show promising potential of GPR30 agonism as a therapeutic strategy against atherogenesis as treatment with G1 rescued OSS-induced oxidative stress by reducing generation of ROS and 4-HNE, reduced OSS-induced expression of TNF-α and IL-6, prevented OSS-induced attachment of monocytes to endothelial cells by reducing expression of VCAM-1 and E-selectin, and rescued OSS-induced reduced KLF2 expression through the ERK5 pathway.

## RESULTS

### OSS reduces expression of GPR30

**Figure 1:**
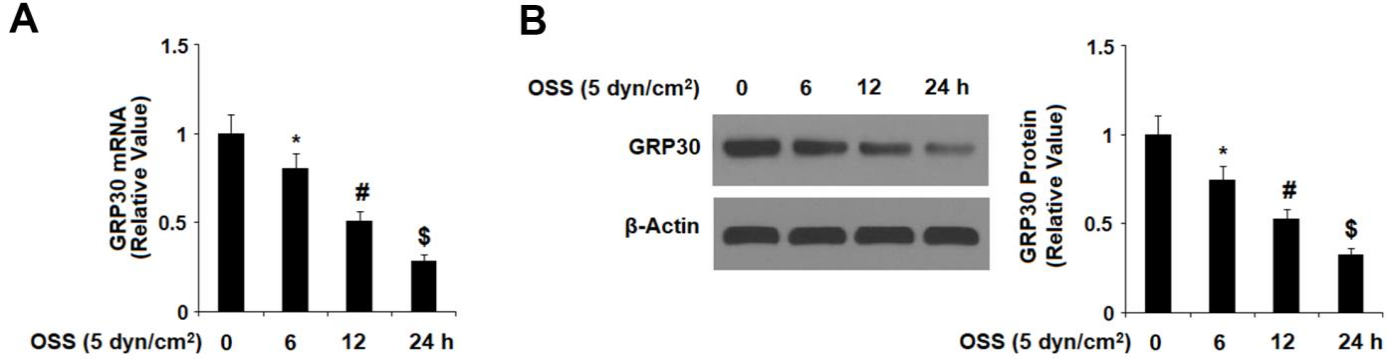
**Oscillatory shear stress (OSS) reduced the expression of GPR30 in human aortic endothelial cells (HAECs) in a time-dependent manner.** HAECs were exposed to OSS (5 dyn/cm^2^) for various periods of times (6, 12, 24 h). (**A**). Expression of GPR30 at the mRNA level; (**B**). Expression of GPR30 at the protein level (*, #, $, P<0.01 vs. previous column group, N=5).

**Figure 2:**
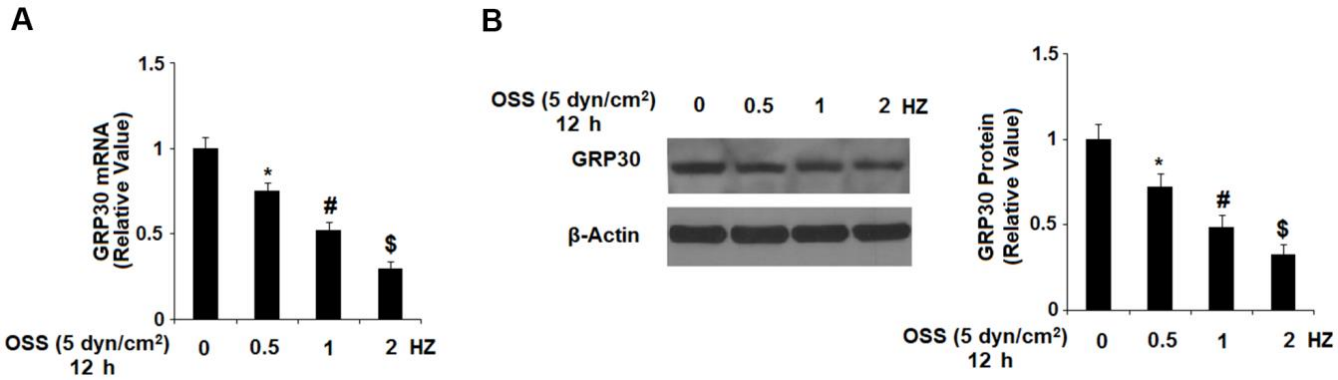
**OSS reduced GPR30 expression in a frequency-dependent manner.** HAECs were exposed to OSS (± 5 dyn/cm^2^) at frequencies of 0.5, 1.0, and 2.0 Hz for 12 h. (**A**). Expression of GPR30 at the mRNA level; (**B**). Expression of GPR30 at the protein level (*, #, $, P<0.01 vs. previous column group, N=5).

First, we investigated the effects of OSS on the expression of GPR30 in HAECs. Our results indicate that exposure to OSS downregulated the expression of GPR30 in a consistent time-dependent manner. As shown in Figure 1, exposure to ± 5 dyn/cm^2^ OSS for 6, 12, and 24 h reduced the expression of GPR30 by roughly 21.2%, 50.5%, and 74.3% at the mRNA level, and roughly 24.3%, 48.2%, and 68.6% at the protein level, respectively. To determine whether different rates of OSS differentially regulate GPR30 expression, HAECs were exposed to OSS (± 5 dyn/cm^2^) at frequencies of 0.5, 1.0, and 2.0 Hz for 12 h. The results in Figure 2 demonstrate that exposure to OSS (± 5 dyn/cm^2^) at frequencies of 0.5, 1.0, and 2.0 Hz reduced the expression of GPR30 by approximately 24.5%, 47.9%, and 70.1% at the mRNA level, and approximately by 27.8%, 51.2%, and 67.4% at the protein level, respectively.

### GPR30 mediates OSS-induced oxidative stress

**Figure 3:**
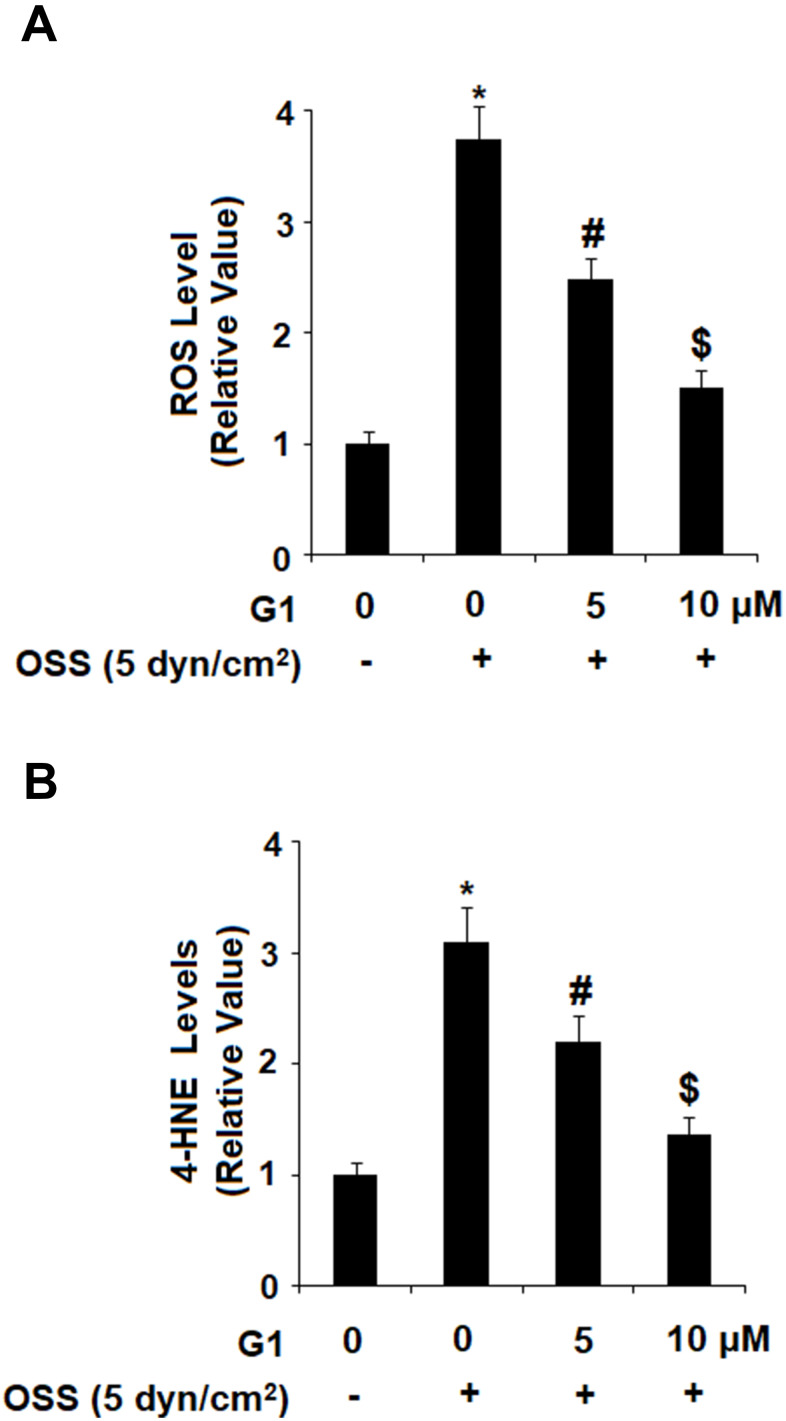
**Agonism of GPR30 using its specific agonist G1 suppressed oscillatory shear stress (OSS)-induced oxidative stress in HAECs.** HAECs were exposed with OSS (5 dyn/cm^2^) in the presence or absence of 5, 10 μM G1 for 24 h. (**A**). Levels of intracellular reactive oxygen species (ROS) were determined by DCFH-DA staining; (**B**). Levels of intracellular 4-hydroxynonenal (4-HNE) were determined by immunostaining (*, #, $, P<0.01 vs. previous column group, N=5).

Oxidative stress plays an important role in the initiation and progression of atherosclerosis. To determine the level of OSS-induced oxidative stress, HAECs were exposed to OSS (± 5 dyn/cm^2^) in the presence or absence of 5 and 10 μM G1 for 24. Then, generation of ROS was measured by DCFH-DA staining and expression of 4-hydroxynonenal (4-HNE) was measured by immunostaining. As demonstrated by the results in Figure 3A, OSS increased production of ROS to roughly 3.7- baseline, which was reduced to only 2.5- and 1.5-fold in a dose-dependent manner by the presence of the two doses of OSS. Immunostaining results in Figure 3B indicated that the level of intracellular 4-HNE was increased to approximately 3.1-fold by OSS (± 5 dyn/cm^2^). However, treatment with the two doses of G1 reduced 4-HNE expression to only 2.2- and 1.3-fold, respectively, indicating a strong ability of GPR30 to modulate OSS-induced oxidative stress.

### GPR30 agonism reduces OSS-induced expression of proinflammatory cytokines

**Figure 4:**
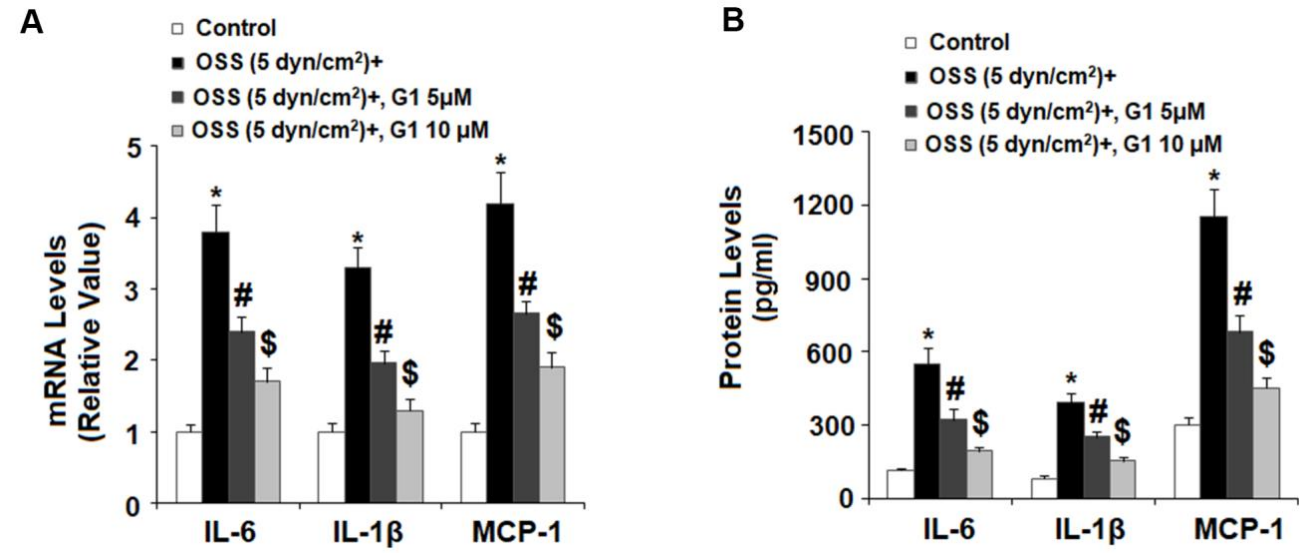
**Agonism of GPR30 using its specific agonist G1 suppressed oscillatory shear stress (OSS)-induced expression and secretion of pro-inflammatory cytokines IL-6, IL-1β, and MCP-1 in human aortic endothelial cells (HAECs).** HAECs were exposed with OSS (5 dyn/cm^2^) in the presence or absence of 5, 10 μM G1 for 24 h. (**A**). Expression of IL-6, IL-1β, and MCP-1 at the mRNA level was determined by real time PCR analysis; (**B**). Secretion of IL-6, IL-1β, and MCP-1 at the protein level was determined by ELISA (*, #, $, P<0.01 vs. previous column group, N=5).

Next, we set out to determine the effects of GPR30 agonism on expression of proinflammatory cytokines including IL-6, IL-1β, and MCP-1 induced by OSS. Briefly, HAECs were exposed to OSS (± 5 dyn/cm^2^) in the presence or absence of 5 and 10 μM G1 for 24 h and cytokine expression was determined at the mRNA and protein levels by real time PCR and ELISA, respectively. As shown in Figure 4A, real-time PCR results indicated that OSS increased the mRNA expression of IL-6, IL-1β, and MCP-1 to approximately 3.8-, 3.3-, and 4.2- fold, respectively. However, the introduction of 5 and 10 μM G1 suppressed the increase in mRNA expression of IL-6 to only 2.4- and 1.7-fold, respectively, that of IL-1β to only 1.9- and 1.2-fold, and that of MCP-1 to only 2.6- and 1.9 fold. The results of ELISA in Figure 4B demonstrate that the protein concentrations of IL-6, IL-1β, and MCP-1 were increased from 115.3±9.3, 82.5±9.1, and 302.7±28.1 pg/mL to 551.8±62.8, 392.5±41.2, and 1153.6±110.6 pg/mL. However, treatment with the two doses of G1 reduced the secretion of IL-6 in a dose-dependent manner to only 323.6±41.2 and 193.4±19.1 pg/mL, secretion of IL-1β was reduced to 252.8±23.4 and 153.6±16.7 pg/mL, and secretion of MCP-1 was decreased to 682.7±66.5 and 452.7±44.3 pg/mL, respectively.

### GPR30 agonism prevents OSS-induced adhesion of monocytes to endothelial cells

**Figure 5:**
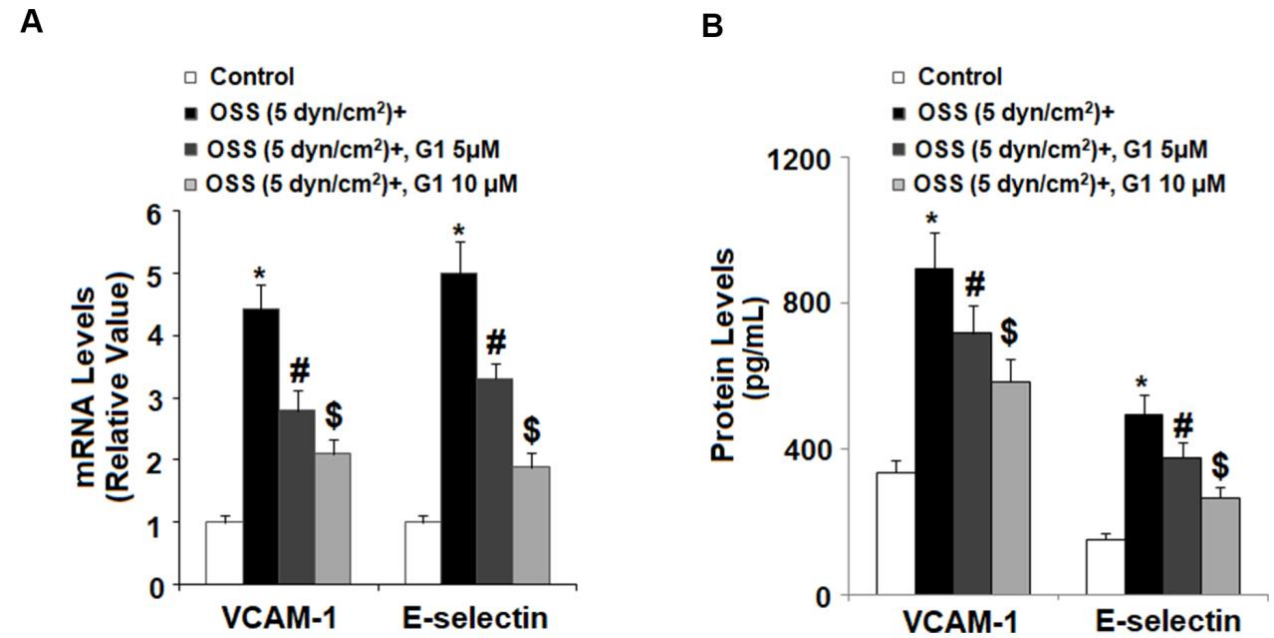
**Agonism of GPR30 using its specific agonist G1 suppressed oscillatory shear stress (OSS)-induced expression of VCAM-1 and E-selectin in human aortic endothelial cells (HAECs).** HAECs were exposed with OSS (5 dyn/cm^2^) in the presence or absence of 5, 10 μM G1 for 24 h. (**A**). Expression of VCAM-1 and E-selectin at the mRNA level; (**B**). Expression of VCAM-1 and E-selectin at the protein level was determined by ELISA (*, #, $, P<0.01 vs. previous column group, N=5).

To determine whether agonism of GPR30 affects adhesion of monocytes to endothelial cells, we first investigated OSS-induced expression of two key adhesion molecules: VCAM-1 and E-selectin by real time PCR and western blot analysis. Briefly, HAECs were exposed to OSS (± 5 dyn/cm^2^) in the presence or absence of 5 and 10 μM G1 for 24 h. As demonstrated in Figure 5A, mRNA expression of VCAM-1 and E-selectin was increased to roughly 4.4- and 5.1-fold baseline at the mRNA level. However, the introduction of 5 and 10 μM G1 suppressed the increase in mRNA expression of VCAM-1 to only 2.7- and 2.1-fold, respectively, and that of E-selectin to only 3.2- and 1.9 fold. Consistently, expression of these two adhesion molecules at the protein level was significantly increased in response to OSS. Treatment with G1 decreased protein expression by more than half (Figure 5B).

**Figure 6:**
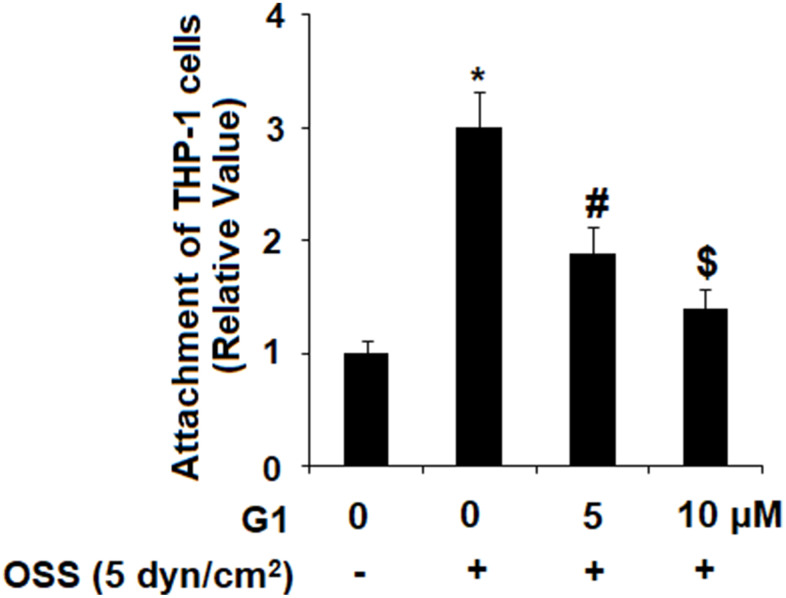
**Agonism of GPR30 using its specific agonist G1 suppressed oscillatory shear stress (OSS)-induced adhesion of THP-1 cells to human aortic endothelial cells (HAECs).** HAECs were exposed with OSS (5 dyn/cm^2^) in the presence or absence of 5, 10 μM G1 for 24 h. Attachment of THP-1 cells to HAECs was determined (*, #, $, P<0.01 vs. previous column group, N=5).

Next, we investigated the effect of GPR30 agonism on adhesion of human monocytic cell line THP-1 cells to endothelial cells by exposing HAECs and THP-1 cells to OSS (± 5 dyn/cm^2^) in the presence or absence of 5 and 10 μM G1 for 24 h. As demonstrated by the results in Figure 6, OSS roughly increased the amount of THP-1 cells bound to HAECs to 3.1-fold, which was reduced by G1 in a dose-dependent manner to 1.9- and 1.3- fold, respectively. Thus, agonism of GPR30 by G1 shows a promising inhibitory effect against attachment of monocytes to endothelial cells induced by OSS.

### GPR30 agonism rescues OSS-induced reduced KLF2 through the ERK5 pathway

**Figure 7:**
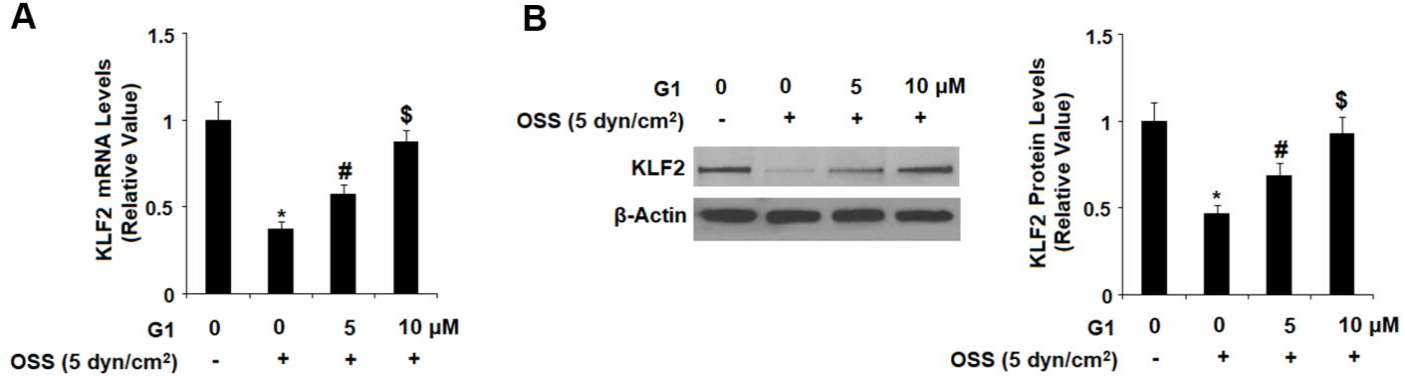
**Agonism of GPR30 using its specific agonist G1 rescued oscillatory shear stress (OSS)-induced reduction of KLF2. HAECs were exposed with OSS (5 dyn/cm^2^) in the presence or absence of 5, 10 μM G1 for 24 h.** (**A**). Expression of KLF2 at the mRNA level was determined by real time PCR analysis; (**B**). Expression of KLF2 at the protein level was determined by western blot analysis (*, #, $, P<0.01 vs. previous column group, N=5).

**Figure 8:**
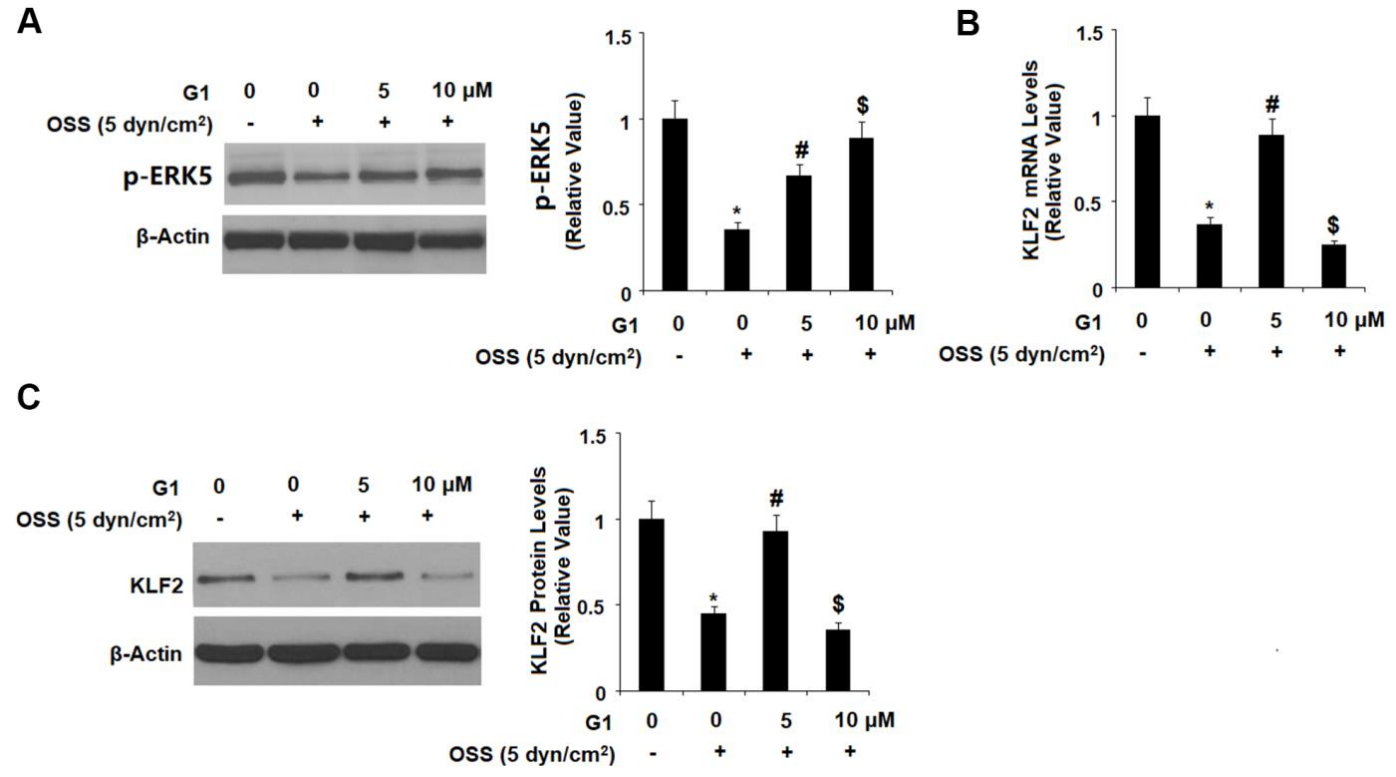
**The effects of G1 on KLF2 expression are mediated by ERK5.** (**A**) HAECs were exposed to OSS (5 dyn/cm^2^) in the presence or absence of 5, 10 μM G1 for 2 h. Phosphorylated levels were measured by western blot analysis; (**B**, **C**). HAECs were exposed to OSS (5 dyn/cm^2^) in the presence or absence of 10 μM G1 or the specific ERK5 inhibitor XMD8-92 (10 nM) for 24 h. The expression of KLF2 at the mRNA level and protein level were determined by real time PCR and western blot analysis, respectively (*, #, $, P<0.01 vs. previous column group, N=5).

Expression of KLF2 by endothelial cells is known to exert protective effects against atherosclerosis, however OSS greatly reduces expression of KLF2 at both the mRNA and protein levels. To determine whether GPR30 plays a role in OSS-induced downregulation of KLF2, HAECs were exposed to OSS (± 5 dyn/cm^2^) in the presence or absence of 5 and 10 μM for 24 h. As shown by the results in Figure 7, OSS reduced expression of KLF2 by roughly 60 %, which was rescued by treatment with G1 in a dose-dependent manner, with the higher dose remarkably restoring KLF2 expression to 88 % at the mRNA level and 93 % at the protein level. Next, we set out to determine whether this effect occurs through the ERK5 pathway. Briefly, HAECs were exposed to OSS (± 5 dyn/cm^2^) in the presence or absence of 5 and 10 μM for 2 h. As shown in Figure 8A, OSS reduced the level of phosphorylated ERK5 by more than half which was rescued by treatment with G1 in a dose-dependent manner. Interestingly, the presence of the specific ERK5 inhibitor XMD8-92 (10 nM) abolished the effects of G1 in the expression of KLF2 at both the mRNA levels (Figure 8B) and protein levels (Figure 8C).

## DISCUSSION

The prevention and treatment of atherosclerosis remains a challenge due the multitude of factors contributing to its pathology. In the present study, we elucidated the role of the estrogen-sensing G-coupled protein receptor GPR30 in atherogenesis-related endothelial dysfunction. Our findings show that agonism of GPR30 by G1 can significantly downregulate OSS-induced oxidative stress, release of proinflammatory cytokines and adhesion molecules thereby preventing adhesion of monocytes to endothelial cells and endothelial dysfunction. Our results demonstrate a significant protective effect of G1 at the concentrations of 5 and 10 μM against OSS-induced insults in HAECs, which is consistent with a previous study showing that administration of G1 (10 μM) displayed the most robust relaxing effect in carotid arteries from both male and female rats by scavenging superoxide [20], whereas Chakrabarti and Davidge [21] found that treatment with G1 attenuates TNF-induced upregulation of ICAM-1 and VCAM-1 in human umbilical vein endothelial cells (HUVECs) at the concentrations of 1 and 3 μM. It should be noted that estrogen signaling in the vascular system is a complex phenomenon involving different receptors and signaling pathways, which can be altered depending on cell type. This could reflect more efficient signaling via G protein-coupled receptors or a greater binding affinity for G1 in HUVECs than in HAECs. We also found that GPR30 plays a role in rescuing downregulation of the cardioprotective transcription factor KLF2 induced by OSS via the ERK5 pathway.

Oxidative stress is a major contributor in numerous disease states and has been cited as the primary cause of endothelial dysfunction [22]. Recent research using GPR30 knockout rats showed that lack of GPR30 increases cardiac oxidative stress as demonstrated by a decrease in the oxidant/antioxidant ratio, increased expression of 4-HNE as well as various oxidative stress-related genes [23]. Another study showed that agonism of GPR30 may exert a neuroprotective effect against oxidative stress induced by chronic cold exposure [24]. Our findings provide further evidence of an anti-oxidative stress role of GPR30 in atherosclerosis as treatment with G1 inhibited generation of ROS and expression of 4-HNE induced by OSS. Chronic inflammation greatly contributes to atherogenesis and strategies to resolve inflammation by inhibiting the release of proinflammatory cytokines including TNF-α and IL-6 are considered a promising treatment path. The role of estrogen as an anti-inflammatory and anti-cardiovascular disease agent has been thoroughly investigated, but it is not yet clear how individual estrogen receptors such as GPR30 participate in this activity. A study using G1 demonstrated that agonism of GPR30 could reduce TNF-α-induced expression of intercellular adhesion molecule 1 (ICAM-1) and VCAM-1 in human umbilical vein endothelial cells [21]. G1 has also been shown to reduce expression of TNF-α and IL-6 in human blood mononuclear cells [25]. In the present study, we demonstrated that GPR30 may play a role in modulating chronic inflammation induced by hydromechanical forces, namely OSS, by reducing expression of TNF-α and IL-6 in HAECs. These findings shed additional light on the potential role of GPR30 as an anti-inflammatory target in cardiovascular various diseases including atherosclerosis.

Endothelial dysfunction and attachment of monocytes to endothelial cells is critical to the process of atherogenesis. Therapies to prevent attachment of monocytes to endothelial cells via downregulation of adhesion molecules such as VCAM-1 and E-selectin are a valuable approach. Various studies have demonstrated the ability of G1 to downregulate expression of VCAM-1 expression induced by TNF-α [26, 27]. In the present study, we demonstrate that GPR30 can downregulate expression of VCAM-1 as well as E-selectin induced by OSS, thereby significantly reducing the number of monocytes that adhere to endothelial cells. Thus, the triple ability of GPR30 to regulate oxidative stress, inflammatory response, and secretion of adhesion molecules induced by OSS suggests that it may be a valuable treatment target for atherosclerosis. Finally, we looked at the effect of GPR30 agonism on expression of KLF2, a key protector against cardiovascular disease. KLF2 is known to be upregulated in response to shear stress and to induce activation of numerous downstream signaling pathways that exert protective effects against atherosclerosis [28, 29]. However, expression of KLF2 is flow-dependent, and OSS had been shown to induce transient upregulation of KLF2 followed by prolonged suppression [30]. To our knowledge, there has been little to no research on the role of GPR30 in OSS-mediated downregulation of KLF2. In the present study, we found that OSS exerted an inhibitory effect against KLF2 which could be rescued by agonism of GPR30 in a dose-dependent manner. We also demonstrate that this effect is mediated through the ERK5 pathway. OSS had a similar inhibitory effect against KLF2 to that of the specific ERK5 inhibitor XMD8-92, and that this could be almost completely ameliorated by agonism of GPR30 using G1.

The underlying mechanism whereby OSS induces endothelial dysfunction is now becoming increasingly more apparent, involving several procoagulant signaling pathways, including nuclear factor kappa B (NF-κB), the central regulator of inflammation signaling. Crosstalk between the KLF2 and NF-κB pathways has been reported in previous studies. Indeed, KLF2 can inhibit NF-κB activation by competing for the common coactivators p300 and PCAF [31]. NF-κB can also inhibit the expression of KLF2 in the presence of TNF-α, which is dependent on the nuclear translocation of p65 [32]. Interestingly, a recent study demonstrated that activation of GPR30 exerts acute neuroprotective effects by inhibiting TLR4/NF-κB-mediated inflammation in microglia [33]. Importantly, it has been reported that agonism of GPR30 with G1 mitigates H2O2-induced endothelial inflammation through suppressing NF-κB activity [27]. Therefore, it is possible that the interplay between NF-κB and KLF2 plays a role in the protective effects of GPR30 and G1 against OSS-induced insults in HAECs. Future investigations will be helpful for clarifying the underlying molecular mechanisms.

Recent work has revealed that endothelial GPR30 mediates estrogen mediated anti-inflammatory effects in vascular cells [34]. GPR30 activation mainly promotes estrogen-dependent nitric oxide formation and vasodilation [35, 36]. GPR30 is also involved in homocysteine induced protection of endothelial cell in mouse model of atherosclerosis [37]. Mechanistic study shows GPR30 protects endothelial cells through the activation of eNOS and Akt and may contribute to vascular angiogenesis [38]. Our findings show GPR30 activation is a protective force in atheroprone flow induced vascular stress. All these facts indicate GPR30 signaling play a critical role in vascular functions and stress induced protection. Preclinical study shows GPR30 agonist G-1 therapy indeed improves cardiac mass and atherogenic cardiovascular risks in diabetic rats [39]. G-1 and other GPR30 ligands have been proposed as the potential compounds to treat coronary artery diseases [40]. Therefore, the activation of GPR30 by its ligands to benefit vascular health is an attractive choice in future therapeutic trials.

### Limitations

The major limitation of our study is that all the experiments were conducted *in vitro* in cultured HAECs under OSS. Although our method to induce OSS by the orbital cone is a convenient approach for investigating endothelial mechanic response, but this approach is not the perfect model to explore the physiological response of GPR30 activation [41]. In the physiological state, the aorta wall is exposed to multiple stimuli in addition to shear stress, therefore potential contribution of other stimuli such as stretch force or chemical stimuli cannot be fully replicated *in vitro*. Oscillatory shear flow is often observed in the regions of aortic arch, bifurcation area of carotid artery and abdominal aorta, which are highly susceptible to the formation of atherosclerosis [42]. Oscillatory shear flow generated *in vitro* would not be the ideal environment to study the atherosclerosis.

### Future directions

Future experiments *in vivo* should be performed to assess the molecular pathways of vascular mechanic response of GPR30 activation and the potential treatment effectiveness of G-1 on plaque burden in atherosclerosis susceptible region.

## CONCLUSIONS

Our study demonstrates a prominent role of GPR30 activation on protecting endothelial dysfunction. GPR30 activation suppresses oscillatory shear stress- induced oxidative stress, and limits the release of inflammatory cytokines and the adhesion of monocytes to endothelial cells via promoting the ERK5/KLF2 signaling. Further investigation *in vivo* is necessary to elucidate the role of GPR30 in the modulation of atherosclerosis.

## MATERIALS AND METHODS

### Cell culture, shear stress exposure, and treatment

Human aortic endothelial cells (HAECs) were obtained from Lonza (Basel, Switzerland) and the human monocytic leukemia THP-1 cell line was purchased from ATCC (USA). HAECs were maintained using a Microvascular Endothelial Cell Growth Medium-2 BulletKit from Lonza, USA. THP-1 cells were cultured in DMEM media containing 10 % fetal serum. HAECs, grown to 90 % confluency in 10 cm plates, were exposed to a Teflon cone with directional changes of flow at 1 Hz cycle (± 5 dyn/cm^2^) for OSS in the presence or absence of 5 and 10 μM G1 for 24 h.

### Quantitative real time PCR analysis

After the stimulation, intracellular RNA was isolated from HAECs using a high pure RNA isolation kit (#11828665001, Roche, Switzerland). The concentration and quality of isolated RNA was assessed using a NanoDrop spectrophotometer. Equal amounts of RNA (2 μg) from each group were used to produce cDNA using iScript reverse transcription Supermix (Thermo Fisher Scientific, USA). Expression of target genes was determined using SYBR Green-based real time PCR analysis with SYBR Green Master Mix (Applied Biosystems, USA) on an ABI 7500 platform. The samples run as triplicate. Relative mRNA expression was calculated using the 2^−ΔΔCt^ method with glyceraldehyde-3-phosphate dehydrogenase (GAPDH) as a reference gene. The primers were listed in Table 1.

**Table 1:** Real time PCR primers.

**Target**	**Accession no.**	**Oligo**	**Sequence**
**IL-1β**	M15131	Forward	5’-GCACACCCACCCTGCA-3’
		Reverse	5’-ACCGCTTTTCCATCTTCTTCTT-3’
**IL-6**	NM_031168	Forward	5’-TCCAGAAACCGCTATGAAGTTC-3’
		Reverse	5’-CACCAGCATCAGTCCCAAGA-3’
**MCP-1**	NM_011333	Forward	5’-GTTGGCTCAGCCAGATGCA-3’
		Reverse	5’-AGCCTACTCATTGGGATCATCTTG-3’
**VCAM-1**	NM_011693.3	Forward	5’-GATGTAAAAGGAAAAGAACATAACAAGAAC-3’
		Reverse	5’-GATGGCAGGTATTACCAAGGAAGA-3’
**E-selectin**	NP_000441	Forward	5’-GGGAATTCGT GTGACCCTGGCTTC-3’
		Reverse	5’-GGAAGCTTGGAATAGGAGCACTCC-3’
**GPR30**	NM_001505	Forward	5’-CCACGCTCAAGGCAGTCATA-3’
		Reverse	5’-GCACTGCTGAACTTGACATCTGA-3’
**GAPDH**	NM_008084.2	Forward	5’-GACGGCCGCATCTTCTTGT-3’
		Reverse	5’-CAGTGCCAGCCTCGTCCCGTAGA-3’

### Western blot analysis

After the indicated stimulation, cell lysates were prepared from HAECs using a commercial cell lysis containing protease and phosphatase inhibitor cocktail (#5872, Cell Signaling Technology, USA). Protein concentration was measured using the BCA method (Thermo Fisher Scientific, USA). Protein from each group (20 μg) was subjected to 10-12% sodium dodecylsulphate polyacrylamide gel electrophoresis (SDS-PAGE). Separated proteins were then transferred to a PVDF membrane (Bio-Rad, USA), then blocked with 5 % fat skim milk in TBST. The PVDF membrane was then sequentially probed with primary antibodies overnight at 4° C and horseradish peroxidase (HRP)-conjugated secondary antibody for 1 h at RT. Blots were developed with enhanced chemiluminescence (ECL) western blot substrate (Thermo Fisher Scientific, USA) and exposed on X-ray film (#4741019291, Fujifilm). The following antibodies were used in this study: GPR30 (1:1000, #ab39742, Abcam, USA), KLF2 (1:1000, #ab139699, Abcam, USA), p-ERK5 (1:1000, #3371, Cell Signaling Technology, USA), β-actin (#1:10000, #4970, Cell Signaling Technology, USA), anti-rabbit IgG, HRP-linked secondary antibody (1:3000, #7074, Cell Signaling Technology, USA); anti-mouse IgG, HRP-linked antibody (1:3000, #7076, Cell Signaling Technology, USA).

### Determination of reactive oxygen species (ROS)

After the stimulation, intracellular levels of ROS in HAECs were assessed using 2',7'-dichlorodihydrofluorescein diacetate (DCFH-DA) (Sigma-Aldrich, USA). HAECs were exposed to OSS (± 5 dyn/cm^2^) in the presence or absence of 5 and 10 μM G1 for 24 h. After 3 gentle washes with PBS, cells were probed with 5 μM DCFH-DA in Hank’s balanced salt solution (HBSS) for 30 min in darkness. Fluorescence signals were visualized with an inverted fluorescence microscope (Zeiss, Germany).

### 4-hydroxy-2-nonenal (4-HNE)

The levels of 4-HNE in HAECs were measured by immunofluorescence to reflect the patterns of lipid peroxidation. After the stimulation, HAECs were fixed in 4 % paraformaldehyde for 10 min at RT and permeabilized with 0.4 % Triton X-100 in TBST for 15 min on ice. Cells were blocked with 5 % BSA and 2.5 % FBS for 1 h at RT. After that, cells were incubated with the primary anti-4-HNE monoclonal antibody (ab48506, Abcam, USA) for 2 h. After 3 washes with TBST, cells were probed with the Alexa-594 conjugated secondary antibody (Thermo Fisher Scientific, USA) for 1 h at RT. Fluorescence signals were visualized using an inverted fluorescence microscope (Zeiss, Germany).

### Adhesion of THP-1 monocytes to HAECs

HAECs were exposed to OSS (± 5 dyn/cm^2^) in the presence or absence of 5 and 10 μM G1 for 24 h. THP-1 cells (5-6 × 10^6^ cells/ml) were labeled with 10 μM calcein AM (Sigma-Aldrich, USA) for 45 min at 37° C. After the stimulation, THP-1 cells were added to HAECs at a ratio of 5:1 and incubated for 2 h at 37° C in darkness. Unbound cells were washed away with PBS. Adherent THP-1 cells were observed under a fluorescence microscope (Zeiss, Germany).

### Enzyme linked immunosorbent assay (ELISA)

HAECs were exposed to OSS (± 5 dyn/cm^2^) in the presence or absence of 5 and 10 μM G1 for 24 h. Cell culture media was collected to analyze the secretions of IL-6, IL-1β, and MCP-1. Concentrations of these proteins were assayed by ELISA using commercial kits from R&D Systems in accordance with the manufacturer’s instructions: human IL-6 ELISA kit (#D6050), human IL-1β ELISA kit (#DLB50), and human MCP-1 ELISA kit (#DCP00), human VCAM-1 ELISA kit (#DY809), human E-selectin ELISA kit (#DY724).

### Statistical analysis

All experimental results are expressed as means ± S.E.M. Statistical analysis was performed using SPSS (Version 17). The means of multiple groups were compared by analysis of variance (ANOVA) with post-hoc Tukey HSD test, and the normality of each group was assessed by Shapiro-Wilk test. P<0.05 was considered statistically significant.
